# The role of high load herpes simplex virus in patients with mechanical ventilation: a real hospital acquired viral lung infection needs antiviral therapy?

**DOI:** 10.1186/s13054-020-2815-9

**Published:** 2020-04-07

**Authors:** Heyan Wang, Hangyong He

**Affiliations:** 1 Department of Critical Care Medicine, The Sixth Hospital of Guiyang, Guiyang, Guizhou China; 2 Department of Respiratory and Critical Care Medicine, Beijing Institute of Respiratory Medicine, Beijing Chao-Yang Hospital, Capital Medical University, No. 8 Gongren Tiyuchang Nanlu, Chaoyang District, Beijing, 100020 China

Dear editor,

We read with interest of the article by Lukas Schuierer and colleagues [1] about that in patients with ventilator-associated pneumonia (VAP), antibiotic treatment failure, and high levels of herpes simplex virus (HSV) replication, treatment with acyclovir was associated with longer time to death and improved circulatory and respiratory function. Therefore, they suggested a causative role for high load HSV in the group of patients with antibiotics resistant VAP and an acyclovir therapy in this highly selected patient group. From our point of view, some details about HSV replication in ICU patients with mechanical ventilation (MV) need to be further clarified.

First, why and in whom does high load HSV replication detected in mechanically ventilated patients and should be considered as a causative pathogen? Bruynseels et al. [2] found that 22% of ICU patients had HSV in the throat. HSV recovery from lower respiratory tract (LRT) samples of invasive ventilated patients is probably viral contamination from the upper respiratory tract, as endotracheal intubation, sputum aspiration, and other invasive operations. Furthermore, as the author mentioned [1], HSV reactivation could also present as bronchopneumonitis, which was not a typical VAP that can be recognized by radiological findings [3]. Therefore, all patients with invasive mechanical ventilation and not only VAP patients should be treated as a population at high risk of hospital-acquired HSV infection in LRT and should be evaluated.

Second, how should we monitor HSV load in patients with MV? In Schuierer’s study [1], only one HSV PCR from LRT sample was performed after VAP was suspected. However, monitoring the HSV load routinely per week in MV patients and a trend of increasing HSV load may be more evident for a new infection. And an early throat swab for HSV PCR may be an early suggestion of risk factor for HSV infection in LRT. Furthermore, the concentration of the virus may be influenced by the sample quality from a sputum or BALF and lead to a difficult explanation of viral load by the quantitative PCR. Moreover, acyclovir treatment was associated with a significantly improved circulatory function in Schuierer’s study [1], which may suggest a viremia-related septic shock [4]. Taken together, a standardized protocol for sample collection and sequential LRT HSV monitoring is needed, and a serum test of HSV may be valuable in detecting the development of HSV reactivation and infection in MV patients admitted to ICU.

Finally, in Schuierer’s report [1], the treated patients required lower norepinephrine doses than untreated patients on baseline, and the treated group had a longer ICU stay but not lower mortality. Moreover, they included mainly elderly patients with a median age of 69 and 72 years old. These patients are at a higher risk of dying, but they are more likely to die with the virus than because of the virus. And viral reactivation could more likely be a marker of the disease severity and/or immunosuppression associated with critical illness and therapeutics [5]. Thus, giving antiviral drugs to these patients should therefore be considered cautiously in terms of benefit–risk ratio.

## Authors’ response

Reinhard Hoffmann, Lukas Schuierer

**Fig. 1:**
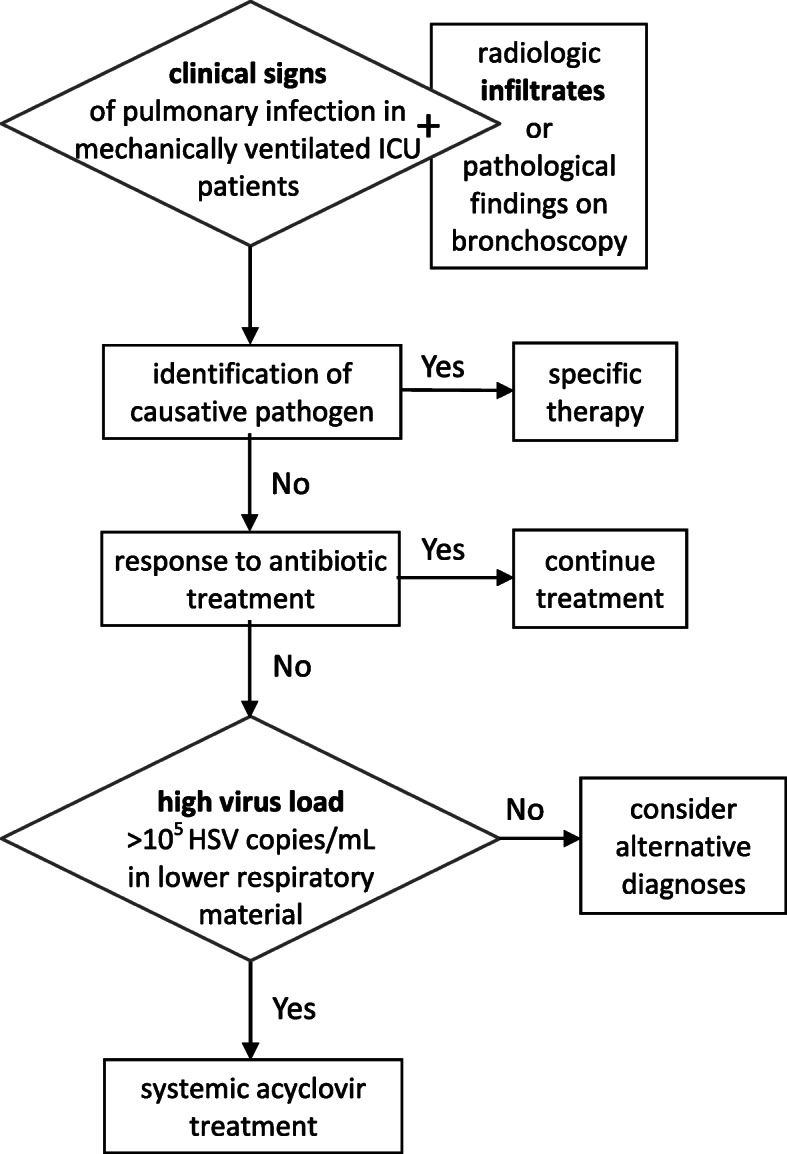
Therapeutic approach (adapted from Forel et al. [9]) *ICU* intensive care unit, *HSV* herpes simplex virus

We thank Drs. Wang and He for their careful evaluation of our paper. First, we agree that detectable herpes simplex virus (HSV) replication is not a rare event in ventilated intensive care unit (ICU) patients [6]. We, however, strictly focused on patients in whom pulmonary infection was unambiguously diagnosed (including cases with normal chest X-ray but pathological findings on bronchoscopy) for which no other cause could be identified, and who do not respond to antibiotic treatment (Fig. 1). This strict selection of patients distinguishes our publication from all previously published studies. We therefore think that HSV is the causative pathogen in our patients. It may well be, however, that it also plays a role in other patient populations which we have not examined. We do not think, however, that widespread screening of ventilated patients is helpful, since it will almost certainly lead to overtreatment of a large proportion of patients which do not have any signs of clinically relevant pulmonary disease—and who, according to a very recent study, will not profit from pre-emptive treatment [7].

Second, as stated above, we do not think that sequential monitoring of patients without clinical evidence of infection is helpful. Moreover, it is not entirely clear to us which type of “blood tests” the authors suggest. In our experience, serum or full blood PCR testing may be performed additionally to the testing of respiratory secretions and would underscore its clinical significance, if positive. We have, however, never really evaluated the diagnostic value of HSV PCR in blood samples—after all, it can be detected in almost 30% of sepsis patients [8]. Serology also may not be helpful given the high rate of latently infected people in the general population, which all have positive serology.

Their final point is related to the first point above—we evaluated only patients with a high likelihood of viral disease before initiation of treatment. Moreover, our result that acyclovir is effective in these patients suggests—a posteriori—that HSV may be the responsible pathogen for pulmonary disease.

Sincerely,

Reinhard Hoffmann

Lukas Schuierer

## Data Availability

Not applicable.
