# Correction to: The role of high load herpes simplex virus in patients with mechanical ventilation: a real hospital acquired viral lung infection needs antiviral therapy?

**DOI:** 10.1186/s13054-020-03094-z

**Published:** 2020-06-25

**Authors:** Heyan Wang, Hangyong He

**Affiliations:** 1 Department of Critical Care Medicine, The Sixth Hospital of Guiyang, Guiyang, Guizhou China; 2 Department of Respiratory and Critical Care Medicine, Beijing Institute of Respiratory Medicine, Beijing Chao-Yang Hospital, Capital Medical University, No. 8 Gongren Tiyuchang Nanlu, Chaoyang District, Beijing, 100020 China

**Correction to: Crit Care (2020) 24:140**

**https://doi.org/10.1186/s13054-020-2815-9**

Following publication of the original article [1], the authors response author reported an error that Fig. 1 was missing in the Author’s response section. The figure is given below.

Another error was identified by the authors‘response authors which reported the references used in the Authors‘response section were incorrect.

The updated Authors‘response and additional references are given below and the changes have been highlighted in **bold typeface**.

**Authors’ response**

Reinhard Hoffmann^2, 3^*, Lukas Schuierer^1, 2, 3^

1: TUM Graduate School, Technical University of Munich (TUM), Germany

2: Institute for Laboratory Medicine and Microbiology, University Hospital Augsburg, Germany

3: Faculty of Medicine, Augsburg University, Germany

We thank Drs. Wang and He for their careful evaluation of our paper. First, we agree that detectable herpes simplex virus (HSV) replication is not a rare event in ventilated intensive care unit (ICU) patients [2]. We, however, strictly focused on patients in whom pulmonary infection was unambiguously diagnosed (including cases with normal chest X-ray but pathological findings on bronchoscopy) for which no other cause could be identified, and who do not respond to antibiotic treatment (Fig. 1). This strict selection of patients distinguishes our publication from all previously published studies. We therefore think that HSV is the causative pathogen in our patients. It may well be, however, that it also plays a role in other patient populations which we have not examined. We do not think, however, that widespread screening of ventilated patients is helpful, since it will almost certainly lead to overtreatment of a large proportion of patients which do not have any signs of clinically relevant pulmonary disease—and who, according to a very recent study, will not profit from preemptive treatment [3]. Second, as stated above, we do not think that sequential monitoring of patients without clinical evidence of infection is helpful. Moreover, it is not entirely clear to us which type of “blood tests” the authors suggest. In our experience, serum or full blood PCR testing may be performed additionally to the testing of respiratory secretions and would underscore its clinical significance, if positive. We have, however, never really evaluated the diagnostic value of HSV PCR in blood samples—after all, it can be detected in almost 30% of sepsis patients [4]. Serology also may not be helpful given the high rate of latently infected people in the general population, which all have positive serology. Their final point is related to the first point above—we evaluated only patients with a high likelihood of viral disease before initiation of treatment. Moreover, our result that acyclovir is effective in these patients suggests—a posteriori—that HSV may be the responsible pathogen for pulmonary disease.

Sincerely,

Reinhard Hoffmann

Lukas Schuierer

**Fig. 1:**
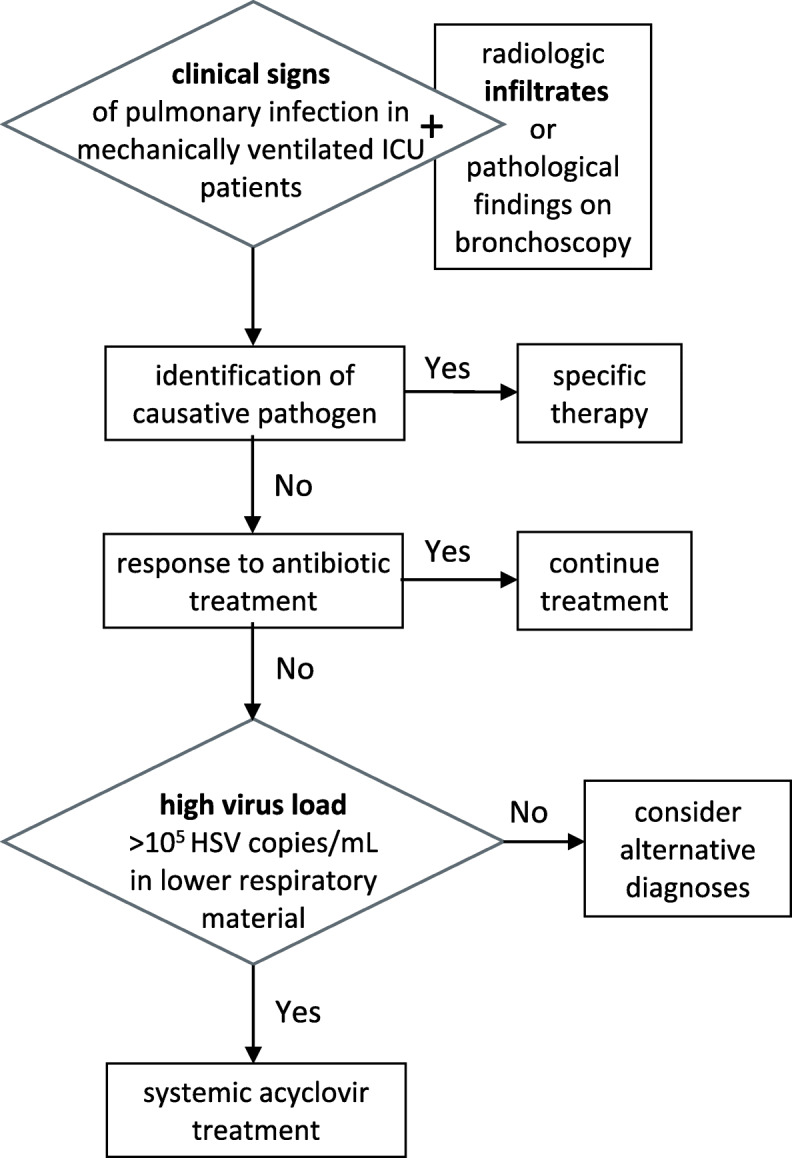
Therapeutic approach (adapted from Forel et al. [5]). *ICU* intensive care unit, *HSV* herpes simplex virus

The original article [1] has been updated.
